# How responding in Spanish affects CAHPS results

**DOI:** 10.1186/s12913-022-08262-1

**Published:** 2022-07-08

**Authors:** Floyd J. Fowler, Philip S. Brenner, Carol Cosenza, Paul D. Cleary

**Affiliations:** 1 Center for Survey Research, University of Massachusetts Boston, 100 Morrissey Blvd., Boston, MA 02125 USA; 2 Department of Sociology, University of Massachusetts Boston, 100 Morrissey Blvd., Boston, MA 02125 USA; 3 Department of Health Policy and Management, Yale School of Public Health, PO Box 208034, New Haven, CT 06520-8034 USA

**Keywords:** Patient experience, Spanish answers in surveys, Quality of health care, CAHPS

## Abstract

**Background:**

The most widely used surveys for assessing patient health care experiences in the U.S. are the Consumer Assessment of Healthcare Providers and Systems (CAHPS) surveys. Studies examining the associations of language and ethnicity with responses to CAHPS surveys have yielded inconsistent findings. More research is needed to assess the effect of responding to CAHPS surveys in Spanish.

**Methods:**

Subjects were patients who had received care at a study community health center in Connecticut within 6 or 12 months of being sent a CAHPS survey that asks about care experiences. The survey included four multi-item measures of care plus an overall rating of the provider. Sampled patients were mailed dual language (English and Spanish) cover letters and questionnaires. Those who did not respond after follow-up mailings were contacted by bilingual interviewers to complete the survey by telephone.

We tested three hypotheses for any observed differences by ethnicity and language:

1. Spanish speakers are more likely than others to choose extreme response options.

2. The semantic meaning of the Spanish translation is not the same as the English version of the questions, resulting in Spanish speakers giving different answers because of meaning differences.

3. Spanish speakers have different expectations regarding their health care than those who answer in English.

Analyses compared the answers on the survey measures for three groups: non-Hispanics answering in English, Hispanics answering in English, and Hispanics answering in Spanish.

**Results:**

The overall response rate was 45%. After adjusting for differences in demographic characteristics and self-rated health, those answering in Spanish gave significantly more positive reports than the other two groups on three of the five measures, and higher than the non-Hispanic respondents on a fourth.

**Conclusions:**

Those answering in Spanish gave more positive reports of their medical experiences than Hispanics and non-Hispanics answering in English. Whether these results reflect different response tendencies, different standards for care, or better care experiences is a key issue in whether CAHPS responses in Spanish need adjustment to make them comparable to responses in English.

## Background

The most widely used surveys for assessing patient health care experiences in the U.S. are the Consumer Assessment of Healthcare Providers and Systems (CAHPS) surveys [1, 2]. Since the inception of the CAHPS program, there has been a commitment to having survey instruments that could be used in multiple languages, including Spanish, as well as in English [3]. Thus, there has been a focus on designing questions that can be interpreted comparably across languages. To achieve that goal, draft survey questions are routinely cognitively tested in Spanish as well as English with monolingual individuals and revised versions are assessed with forward and back translation before final decisions about wording are made [4].

Studies examining the associations of language and ethnicity with responses to CAHPS surveys have yielded inconsistent findings. Although some have found that Hispanic and non-Hispanic whites have similar response patterns [5, 6] if interpretative services are not needed [7], other studies suggest that Hispanic respondents report less positive experiences than non-Hispanic whites [8, 9]. Some studies have found that Hispanic respondents give more positive responses [10, 11]. For example, Setodji et al. [12] analyzed the effect of responding in Spanish and English to CAHPS questions and found that responses in English were more positive for 7 out of 9 items.

These mixed findings are difficult to interpret because, as some authors have noted, response patterns may be affected by differences in care quality, as well as response tendencies related to region of residence, language, and/or acculturation. Several studies, however, suggest that non-Hispanic whites and Hispanic respondents may use the response scales differently [6, 12, 13]. Some authors have found that Hispanic respondents are more likely to use extreme response categories [11, 14]. For example, Hui and Triandis [14] found that Hispanics are more likely to use extreme responses on a 5-point Likert-type response scales than non-Hispanic whites. Marin and colleagues [15] found that the degree to which this was done was associated with acculturation. The longer Hispanic respondents had been in the United States, the lower their tendency to use extreme responses. That tendency was also associated with the level of formal education; respondents with less education are more likely to give extreme responses.

A tendency to give more socially desirable answers has also been observed in Hispanics. This was first reported by Ross and Mirosky [16], who found more than expected socially desirable behaviors and fewer than expected undesirable behaviors reported by Hispanics. Hispanics living in Mexico showed this tendency more than those in the US. Clark, Rogers and Allen [17] found that for six out of nine prevention and screening behaviors, such as getting flu shots, eye exams, and PSA tests, Hispanics responding in Spanish reported significantly higher rates than Hispanics responding in English or non-Hispanic whites. Johnson and colleagues [18] also report that giving the “right” (more socially desirable) answers is more common in Hispanics’ survey responses. Aday and colleagues [19] reported that Hispanics overreported seeing a doctor more than non-Hispanics (who also overreported visits to doctors).

Studies have examined the associations among items and scales, with several suggesting that the associations vary by ethnic groups [20]. For example, some have found that Hispanic respondents give more positive ratings than other respondents, taking into account responses to other questions about health care experiences, which may reflect lower expectations for health care quality [21, 22].

As part of a larger study of patients receiving care in community health centers in Connecticut, we collected survey data that could be used to assess the effect of ethnic background and language on CAHPS answers. The study included many self-identified Hispanic respondents who responded in either English or Spanish.

The purpose of this paper is to compare responses in English and in Spanish to the CAHPS questions. Given the explanations of response patterns suggested by previous studies, we test three hypotheses for any observed differences:

1. Spanish speakers are more likely than others to choose extreme response options.
2. The semantic meaning of the Spanish translation is not the same as the English version of the questions, resulting in Spanish speakers giving different answers because of meaning differences.
3. Spanish speakers have different expectations regarding their health care than those who answer in English.

## Methods

### Sample

The data for this study were collected as part of a larger evaluation of a care coordination program in a group of community health centers [23]. The study selected a stratified random sample of adults (18 years of age or older). One stratum consisted of adults who had at least one visit with their primary care physician in the past 12 months. The other stratum comprised patients who made at least two visits in the past 12 months and were eligible for a care coordination program because of complex health care needs. As part of a methodological study, a subsample of patients was sent a survey with a 6-month reference period, whereas all other patients received a survey with a 12-month reference period [24]. In order to study the effects of ethnicity and language on responses, we restricted this analysis to the respondents for whom ethnicity was ascertained.

### Data collection

A standard CAHPS protocol was used for data collection. An initial survey was mailed to all sampled individuals, along with a cover letter explaining the purposes of the survey and assuring confidentiality and that participation was voluntary. All letters were in Spanish and in English. The questionnaires were “Canadian style”, with both English and Spanish questions in the same questionnaire.

Initial mailings were followed by a postcard reminder to all sampled individuals and then a second mailing of the survey as well as another cover letter to all initial nonrespondents.

Finally, every nonrespondent for whom we had a telephone number was called by trained interviewers who attempted to complete the survey by telephone. Interviewers were available to complete interviews in either English or Spanish.

### Survey

One goal of the survey was to collect data about patient experience in a program emphasizing coordinated care, as in the model of a Patient-Centered Medical Home [23]. The instrument that was used was the standard Clinician & Group CAHPS survey (3.0) [25, 26] with added questions about care coordination [27]. The survey was eight pages long and included 56 questions.

### Measures

Our analyses focus on the effects of Hispanic ethnicity and the language in which questions were answered on the measures that are commonly reported from CAHPS surveys. Four of these measures are multi-item composites that were formed by averaging the responses to questions with valid responses. Each question uses a four-alternative response task. Responses were transformed linearly into a 0 to 10-point scale as follows: never (0), sometimes (3.33), usually (6.67), and always [10]. For each composite, the average was rounded to the nearest integer in the range of 0 to 10. A composite score was treated as missing if the respondent had missing data on more than one of the composite questions. The reference period for answers was either 6 or 12 months, based on the random assignment for the methodological study [24].

The first composite: Getting Timely Appointments, and Information (Timely Appointments), consisted of three questions: “In the last 6/12 months,”

1. when you phoned this provider’s office to get an appointment for care you needed right away, how often did you get an appointment as soon as you needed?”
2. when you made an appointment for a check-up or routine care with this provider, how often did you get an appointment as soon as you needed?”
3. when you phoned this provider’s office during regular office hours, how often did you get an answer to your medical question that same day?”

The second composite: How Well Providers Communicate with Patients (Communication), consisted of four questions: “In the last 6/12 months, how often did this provider”

1. explain things in a way that was easy to understand?
2. listen carefully to you?
3. show respect for what you had to say?
4. spend enough time with you?

The third composite: Providers’ Use of Information to Coordinate Patient Care (Coordination), was measured by three questions: “In the last 6/12 months,”

1. how often did this provider seem to know the important information about your medical history?
2. when this provider ordered a blood test, x-ray, or other test for you, how often did someone from this provider’s office follow up to give you those results?
3. how often did you and anyone in this provider’s office talk about all the prescription medicines you were taking?

The fourth composite:

Helpful, Courteous, and Respectful Office Staff (Office Staff), consists of two questions: “In the last 6/12 months,”

1. how often were clerks and receptionists at this provider’s office as helpful as you thought they should be?
2. how often did clerks and receptionists at this provider’s office treat you with courtesy and respect?

We also analyzed the single item measuring Patients Rating of the Provider (Provider Rating). “Using any number from 0 to 10, where 0 is the worst provider possible and 10 is the best provider possible, what number would you use to rate this provider?”

The survey asked respondents to self-identify as either Hispanic or non-Hispanic. Ethnicity and language were combined into a single variable with three categories using their reported ethnicity and the language in which they responded:

(1) Hispanic ethnicity, answered in Spanish; 2) Hispanic ethnicity, answered in English; (3) non-Hispanic, answered in English.

### Analysis

The first goal was to determine if there were systematic differences related to the language of response. A linear regression model was estimated for each of the five CAHPS measures. The first multivariate models controlled only for ethnicity/language group.

To adjust for patient characteristics that are known to be related to responses on CAHPS surveys [28, 29], a second model included education (high school or less, some college, and college degree or more), sex (male or female), and age (18 to 34, 35 to 44, 45 to 54, and 65 or older) and self-rated health (excellent or very good vs good, fair, or poor) as categorical variables. Race was not included because of substantial missing data. Over half of the respondents reporting Hispanic background did not report a race. All models also included whether the respondents were part of the chronic condition or control stratum, whether they were assigned a 6- or 12-month reference period, and survey mode (phone or mail). Three analyses were then used to better understand the reasons for differences:

#### Use of extreme answers

The first hypothesis posits that those answering in Spanish use extreme response alternatives more of than those answering in English. Although the response option “never” is seldom used in CAHPS surveys, “always” is used frequently and may be used even more readily by respondents answering in Spanish. To examine this hypothesis, we estimated a series of logistic regression models to see if those answering in Spanish used “always” at a different rate from those answering in English. These models estimated the propensity for “always” answers using ethnicity/language as the key independent variable with age, education, and gender as controls.

#### Language

The second hypothesis focuses on a potential translation problem affecting answers in Spanish. The CAHPS team follows a translation process designed to maximize the quality of the translated surveys [4]. First, draft survey questions are cognitively tested in English and in Spanish with monolingual individuals and draft questions are revised as needed. Once a questionnaire is developed, the team uses two translators to each produce a forward translation and then the two forward translations are reviewed (by a separate bilingual reviewer) against each other and compared to the original English survey. However, when we did an independent review of the Spanish wording, with special attention to the “always to never” scale which was used in most of the composite measures, two reviewers wondered if the translation of “usually”, (“La mayoría de las veces”), could lead to slightly different answers. To examine that possibility, we estimated a set of logistic regression models to determine whether those answering in Spanish used “*La mayoría de las veces”* at significantly different rates than English speakers used “usually”. These models estimated the propensity for “usually” answers using ethnicity/language as the key independent variable with age, education, and gender as controls.

#### Expectations

The third hypothesis is that Spanish speakers respond more positively, especially to more subjective questions. This tendency may be due to different standards for care, so that the same experiences would be rated more positively, or a predisposition to give answers they think those conducting the survey will like. To examine this hypothesis, we estimated a linear regression model predicting the overall rating of care, controlling for demographic characteristics, health status, the descriptions of the care they had received as reflected in their answers to the questions in the composites, and the language in which the questions were answered.

All aspects of the study were conducted in accordance with relevant regulations and ethical guidelines.

## Results

There were 3007 usable responses to the original survey, resulting in an overall response rate of 45%, using the American Association for Public Opinion Research response rate 3 (completed cases divided by known eligible cases plus a percentage of cases with unknown eligibility based on the estimated rate of eligibility) [30]. Eighty-six cases of respondents who reported that someone else translated or completed the survey for them were omitted. The analyses reported in this paper were restricted to the 2850 respondents who reported their ethnicity. Of those, 56% of the surveys were returned by mail, with the balance being completed by telephone interview. Eighty-two percent of the respondents had multiple visits and chronic conditions. One thousand and forty-seven (38%) of the respondents identified themselves as Hispanic. Of the 2850 surveys, 563 (20%) were completed in Spanish, which constituted 54% of the responses from those who identified as Hispanics.

Table 1 shows the demographic characteristics and self-rated health of the three analysis groups. Hispanic respondents who responded in Spanish were older than Hispanics responding in English and non-Hispanic respondents. They had less formal education than either of the other two groups, and they reported poorer health. However, the poorer reported health was mainly due to Spanish speakers choosing the Spanish translation of “fair” (*regular*) more often than English speakers chose “fair.” They also selected “very good” and “good” less often.

**Table 1:** Demographic characteristics and self-rated health by reported ethnicity (Hispanic or Non-Hispanic) and survey language (English or Spanish)

	Group 1 Non-Hispanic English		Group 2 Hispanic English		Group 3 Hispanic Spanish		X^**2**^ Group 1 Group 3	X^**2**^ Group 2 Group 3
	Freq.	Percent	Freq.	Percent	Frequency	Percent		
Age								
18–34	156	8.7	98	20.3	34	6.1		
35–44	226	12.6	114	23.6	71	12.8		
45–54	577	32.1	150	31.0	160	28.7		
55–64	597	33.2	91	18.8	154	27.7		
65+	243	13.5	31	6.4	138	24.8		
Total	1799	100.0	484	100.0	557	100.0	42.5***	120.8***
Education								
High school or less	1053	59.1	325	67.8	478	87.2		
Some college	547	30.7	121	25.3	45	8.2		
College degree or more	183	10.3	33	6.9	25	4.6		
Total	1783	100.0	479	100.0	569	100.0	149.2***	60.7***
Gender								
Female	1066	59.1	321	66.3	365	64.8		
Male	737	40.9	163	33.7	198	35.2		
Total	1803	100.0	484	100.0	563	100.0	5.8*	0.3
Race								
White	1290	71.5	131	27.1	250	44.4		
Black	322	17.9	28	5.8	18	3.2		
Multi, Other race	191	10.6	39	8.1	13	2.3		
Race missing^a^	0	0.0	286	59.1	282	50.1		
Total	1803	100.0	484	100.0	563	100.0	> 999.0***	46.7***
Self-rated health								
Excellent	92	5.1	46	9.5	57	10.3		
Very good	245	13.7	66	13.7	57	10.3		
Good	572	31.9	169	35.1	122	21.9		
Fair	625	34.9	142	29.5	254	45.7		
Poor	259	14.4	59	12.2	66	11.9		
Total	1793	100.0	482	100.0	556	100.0	51.0***	36.4***

Note: ***p ≤ .001; **p ≤ .01; *p ≤ .05

^a^Many respondents reporting being Hispanic or Latino did not report a race

Table 2 shows the means of the five CAHPS measures for non-Hispanics responding in English, Hispanics responding in English, and those responding in Spanish. For each dependent variable, the top row shows the unadjusted means, and the second row shows marginal means based on output from linear regression models adjusted for demographic differences, self-rated health, and sample and survey characteristics. Before adjustment, four of the measures (but not coordination) showed those responding in Spanish gave significantly more positive responses (*p* < .001) than non-Hispanic respondents and than Hispanics who responded in English. There were no statistically significant differences in the unadjusted means between Hispanics who responded in English and non-Hispanics.

**Table 2:** Adjusted^a^ and unadjusted mean composite scores and provider ratings by language of answers and reported ethnicity (Hispanic or Non-Hispanic)

						Group comparison				
		Group 1 Non-Hispanic, English	Group 2 Hispanic, English	Group 3 Hispanic, Spanish		Group 1 v. Group 2	Group 1 v. Group 3		Group 2 v. Group 3	
Dependent variable (Composites and rating)	Estimate	Mean (SE)	Mean (SE)	Mean (SE)	N	t	t		t	
Timely Appointments	Unadjusted	6.89 (.08)	6.74 (.15)	7.59 (.15)	2033	0.94	4.15	***	4.10	***
	Adjusted	6.91 (.08)	6.89 (.15)	7.38 (.15)	1993	0.13	2.71	**	2.31	*
Communication	Unadjusted	8.56 (.05)	8.53 (.10)	8.95 (.09)	2838	0.25	3.58	***	3.00	**
	Adjusted	8.56 (.05)	8.63 (.10)	8.89 (.10)	2782	0.56	2.87	**	1.81	
Coordination	Unadjusted	7.45 (.06)	7.52 (.12)	7.44 (.12)	2767	0.50	0.05		0.44	
	Adjusted	7.46 (.06)	7.62 (.12)	7.30 (.12)	2717	1.12	1.20		1.86	
Office staff	Unadjusted	7.81 (.06)	7.65 (.12)	8.56 (.11)	2823	1.20	5.82	***	5.53	***
	Adjusted	7.84 (.06)	7.82 (.12)	8.37 (.11)	2775	0.15	4.04	***	3.31	***
Provider rating	Unadjusted	8.45 (.05)	8.53 (.10)	9.16 (.09)	2828	0.71	6.77	***	4.70	***
	Adjusted	8.45 (.05)	8.65 (.10)	9.04 (.09)	2773	1.73	5.37	***	2.83	**

Note: *p ≤ .05; **p ≤ .01; ***p ≤ .001

^a^Models adjust for education, sex, age, self-rated health, sample stratum, survey reference period, and survey mode

After score adjustment, Hispanics responding in Spanish give more positive responses regarding Timely Appointments, Office Staff and Provider Rating compared to Hispanics responding in English and non-Hispanics. There remained no statistically significant differences between Hispanics who responded in English and non-Hispanics. In the adjusted analyses, those responding in Spanish still had the highest score on the Communication composite, but only the comparison with non-Hispanics was statistically significant (*p* < .01).

Hypothesis 1 posited that Hispanics who answered in Spanish are more likely to use extreme response categories. In Table 3, we present the rates at which the language groups gave the extreme response “always.” In Tables 3 and 4, the right two columns show the number of significant differences between those answering in Spanish and the Hispanic and Non-Hispanic respondents who answered in English. Of the variables compared, yielding 36 comparisons between those answering in Spanish vs those answering in English, there were 14 statistically significant results in the rates of answering “always”. Of those, 10 had the Spanish speakers using “always” less frequently, and four had the Spanish speakers using “always” more often. Thus, the data in Table 3 do not support the idea that Spanish-speaking respondents are more likely to choose the most extreme response than others.

**Table 3:** Comparison of frequency of “always” responses to 18 questions used in composite measures by reported ethnicity (Hispanic or Non-Hispanic) and language

Group A	Group B	Number of comparisons	Number of sig. Diff	Number of significant differences with Group B more than Group A	Number of significant differences with Group A more than Group B
Non-Hispanic, English	Hispanic, English	18	1	1	0
Non-Hispanic, English	Hispanic, Spanish	18	7	3	4
Hispanic, English	Hispanic, Spanish	18	7	1	6

**Table 4:** Comparison of frequency of “usually” responses to 18 questions used in composite measures by reported ethnicity (Hispanic or Non-Hispanic) and language of answers

Group A	Group B	Number of comparisons	Number of sig. Diff	Number of significant differences when Group B more than Group A	Number of significant differences when Group A more than Group B
Non-Hispanic, English	Hispanic, English	18	4	0	4
Non-Hispanic, English	Hispanic, Spanish	18	8	1	7
Hispanic, English	Hispanic, Spanish	18	5	3	2

Hypothesis 2 stated that the translation of “usually” may be imprecise and lead to different answers in Spanish than in English. In Table 4, of the 18 comparisons of Hispanics answering in English and those answering in Spanish, there were only five statistically significant differences in the rates at which they used “usually” or “*La mayoría de las veces.*” That does not suggest that there is much of a difference in answers that we can attribute to the translation of “usually.”

Hypothesis 3 posited that those answering in Spanish give more positive answers on average, especially on more subjective questions, either because they feel more positively about the medical care experience, or they want to give more positive answers for other reasons. The CAHPS responses used to form the composite scores are based on questions that ask respondents to report on whether something did or did not happen; often referred to as a “report” question. In CAHPS there is an emphasis on such questions to minimize differences due to different expectations [3]. The rating question (rating of provider on a 0–10 scale), however, is purely subjective and one would expect response tendencies to be more pronounced for the rating than the report questions. Thus, we estimated a linear regression predicting the overall rating of the provider from reports about the quality of care. Estimates presented in Table 5 suggest that, after adjusting for demographic and health differences, sample and survey characteristics, as well as the composite scores, those answering in Spanish gave significantly more positive ratings of their medical care than either of the groups answering in English even after considering any differences in their descriptions of their experiences getting medical care at those providers’ offices. Hispanics answering in English also gave significantly higher ratings of their providers than non-Hispanics, but those answering in Spanish were the most positive.

**Table 5:** Predicted provider rating for language ethnicity groups with adjustments for CAHPS composites and controls^#^

	Predicted Rating	P < Group 3
**Group 1: Non-Hispanic, English**	8.53	***
**Group 2: Hispanic, English**	8.67	*
**Group 3: Hispanic, Spanish**	8.88	N/A
**Change in outcome per 1-point increase in:**	coefficient	P
**Timely Appointments**	0.02	ns
**Communication**	0.65	***
**Coordination**	0.14	***
**Office staff**	−0.03	*

Note: ***p ≤ .001; *p ≤ .05

# Models adjust for education, sex, age, self-rated health, sample stratum, survey reference period, and survey mode

## Discussion

Patients answering in Spanish gave more positive reports about their experiences with medical care than those answering in English, including respondents who self-identified as Hispanic. There are no universally accepted methods for assessing the practical significance of differences in CAHPS scores, although multiple approaches have been proposed [31]. These include indexing by the distribution of patient experience measures or indexing measures against an external anchor, such as the likelihood of disenrollment [32]. Quigley and colleagues have noted that some have used a threshold of 1 point for small and 3 points for medium on a 0–100 score range [33]. Using that standard, the language and ethnicity differences reported in Table 2 would not be considered substantively important. They are suggestive of systematic differences, however, that might be larger in different areas and/or with different ethnic groups.

In this study, we tested three hypotheses for any observed differences:

1. Spanish speakers are more likely than others to choose extreme response options.
2. The semantic meaning of the Spanish translation is not the same as the English version of the questions, resulting in Spanish speakers giving different answers because of meaning differences.
3. Spanish speakers have different expectations regarding their health care than those who answer in English.

The analyses do not support the first two hypotheses about such differences. While others report a tendency for Spanish speakers to use extreme responses more than English speakers, in this study, the extreme response that is most used in CAHPS questions, “always,” was chosen less often by Spanish speakers than English speakers. Overuse of “always” is not the reason for the higher ratings. In addition, concerns about the translation of “usually” as an explanation for differences in reporting were not supported. The three significant differences in the rates of Hispanics picking “usually” in Spanish and English could not account for the differences in quality scores observed.

There were differences between groups in reported health, but the poorer reported health was mainly due to Spanish speakers choosing the Spanish translation of “fair” (*regular*) more often than English speakers chose “fair” (they also selected “very good” and “good” less often). This may be an artifact of different interpretations of “fair” in English and Spanish. There were no significant differences among the three groups in their gender distributions.

We did find, however, that even after we controlled for demographic differences and the composite scores describing their experience with the providers’ offices, Hispanic respondents gave higher provider ratings than non-Hispanics, and Hispanic respondents answering in Spanish gave still more positive ratings of their providers. This pattern is consistent with the notion that there is something about the process of acculturation from a Spanish-speaking country into American life that is associated with the way the CAHPS questions are answered, leading to less positive results.

Another possible explanation for these results is a higher likelihood of Spanish speakers to provide answers to an interviewer than on a mail survey. Such a difference in response propensity between modes could yield higher ratings for Spanish speaking respondents because responses to interviewers have sometimes been found to be more positive than self-administered responses. However, Spanish speakers were no more likely to have responded to an interviewer than those responding in English and mode of response had essentially no effect on the answers, so it is not a factor in our findings.

Two possible explanations for these differences can be found in the literature. First, some have posited that those who are newer to the United States tend to give socially desirable answers, the answers that they think researchers want to hear [16–18]. Second, those who are relatively new to the United States expect less from the health care system and, hence, might give higher scores to similar patient experiences [21, 34]. Irrespective of which explanation is correct, these findings suggest a potential need to adjust for respondent language when making comparisons across providers or groups. If one wants fair comparisons, it is important to compare what one would expect a units’ score to be with comparable patient populations [28, 29, 35].

However, there are concerns about using such adjustments without better understanding the reasons for the effects we observed. For example, if the community health centers from which these samples were drawn were doing a particularly good job of serving Spanish speakers, their efforts should be fully reflected in their CAHPS results. Moreover, although the study included a large sample of Hispanic respondents, some of whom responded in English and some of whom responded in Spanish, the study was conducted in only one state, and it is possible that the results will not generalize to other areas where there are different Hispanic populations with different cultural backgrounds and degrees of acculturation.

There are several potential limitations of these analyses. Although the response rate was consistent with comparable surveys, there were a substantial number of non-respondents, and we had limited information about the differences between those who did and did not respond. The study was conducted in a single state in the northeast of the United States and the Spanish speakers in our study may vary, in terms of cultural background, acculturation, and language style from Spanish speaking groups in other regions. Furthermore, because the CAHPS surveys do not collect detailed information about ethnicity, we could not assess variations among subgroups of Spanish speaking respondents.

In conclusion, these data improve our understanding of how responding in Spanish is related to CAHPS answers in one setting. They clearly point to the possible need for adjusting CAHPS results to permit fair comparisons across providers. However, we need more data, from a variety of types of Spanish speakers and across a variety of provider settings, to decide how best to make CAHPS comparisons.

## Data Availability

The datasets used and analyzed for the current study are not publicly available because the project has ended, and funding is not available for documenting and de-identifying the data. In response to a reasonable request, the data used for this study will be made available from the corresponding author.
